# Deep Sequencing Reveals Low Incidence of Endogenous LINE-1 Retrotransposition in Human Induced Pluripotent Stem Cells

**DOI:** 10.1371/journal.pone.0108682

**Published:** 2014-10-07

**Authors:** Hubert Arokium, Masakazu Kamata, Sanggu Kim, Namshin Kim, Min Liang, Angela P. Presson, Irvin S. Chen

**Affiliations:** 1 Department of Microbiology, Immunology and Molecular Genetics, University of California Los Angeles, David Geffen School of Medicine, Los Angeles, California, United States of America; 2 Korean Bioinformation Center, Korea Research Institute of Bioscience and Biotechnology, Daejeon, South Korea; 3 Department of Biostatistics, University of California Los Angeles School of Public Health, University of California Los Angeles, Los Angeles, California, United States of America; Institute of Medical Biology, Singapore

## Abstract

Long interspersed element-1 (LINE-1 or L1) retrotransposition induces insertional mutations that can result in diseases. It was recently shown that the copy number of L1 and other retroelements is stable in induced pluripotent stem cells (iPSCs). However, by using an engineered reporter construct over-expressing L1, another study suggests that reprogramming activates L1 mobility in iPSCs. Given the potential of human iPSCs in therapeutic applications, it is important to clarify whether these cells harbor somatic insertions resulting from endogenous L1 retrotransposition. Here, we verified L1 expression during and after reprogramming as well as potential somatic insertions driven by the most active human endogenous L1 subfamily (L1Hs). Our results indicate that L1 over-expression is initiated during the reprogramming process and is subsequently sustained in isolated clones. To detect potential somatic insertions in iPSCs caused by L1Hs retotransposition, we used a novel sequencing strategy. As opposed to conventional sequencing direction, we sequenced from the 3′ end of L1Hs to the genomic DNA, thus enabling the direct detection of the polyA tail signature of retrotransposition for verification of true insertions. Deep coverage sequencing thus allowed us to detect seven potential somatic insertions with low read counts from two iPSC clones. Negative PCR amplification in parental cells, presence of a polyA tail and absence from seven L1 germline insertion databases highly suggested true somatic insertions in iPSCs. Furthermore, these insertions could not be detected in iPSCs by PCR, likely due to low abundance. We conclude that L1Hs retrotransposes at low levels in iPSCs and therefore warrants careful analyses for genotoxic effects.

## Introduction

It is now possible to reprogram fully differentiated somatic cells back to the embryonic state by forced expression of certain transcriptional factors such as *OCT4, SOX2, C-MYC* and *KLF4*. These reprogrammed cells, termed ‘induced pluripotent stem cells’ (iPSCs), are capable of unlimited self-renewal and display full pluripotency [1]–[4]. The generation of iPSCs offers a new perspective on the use of stem cells in the regenerative medicine field. Patient-specific iPSCs could then be derived to correct genetic defects in potential cell therapy. However, the safety of these cells has not been thoroughly assessed. Several studies reveal hurdles that must be overcome before any clinical application. In particular, there are concerns about aberrant genomic imprinting, lineage specific differentiation and the potential formation of teratomas and tumors *in* *vivo* [5]–[8]. Another crucial aspect that has been studied is the genomic integrity of these cells. Besides epigenetic aberrations, iPSCs can have abnormal karyotypes, chromosomal aberrations and mutated exomes [9]–[11].

The long interspersed element-1 (LINE-1 or L1) is a retrotransposon of about 6 kb long which replicates itself by a ‘copy paste’ mechanism [12]. L1 is found in more than 500 000 copies in the human genome which are classified in different subfamilies [13], [14]. However, due to diverse mutations, it is estimated that only 80–100 copies of L1 are active in each individual [15]. These active L1s essentially belong to the LINE-1 human specific (L1Hs) subfamily, the youngest and most active L1 subfamily in humans [13], [15], [16]. L1 mobility has been detected in various settings. In the brain, L1 mobility may play a role in neuronal plasticity [17], [18]. However, L1 mobility is also responsible for more than 20 single gene diseases and has also been detected in several types of cancer [19]–[23]. As expected, nearly all L1 insertions in these cases are initiated by the L1Hs subfamily [19]–[23]. L1 is also a potential source of genetic instability and is known to affect gene expression through aberrant splicing and early transcription termination [24]–[28]. Activation of L1 retrotransposition during the reprogramming process or in iPSCs may therefore have detrimental effects.

It has been previously reported that both the expression and the frequency of retrotransposition of L1 are higher in human iPSC clones than in the parental fibroblast cells [29]. However, the retrotransposition frequency results were obtained by the use of an ectopically engineered L1 reporter construct which expressed L1 under either a constitutive promoter or an enhancer. The assay therefore may not reflect the retrotransposition activity of endogenous L1. On the other hand, opposite results were obtained in two other studies. Through whole genome sequencing, others reported that human and mouse iPSCs have stable numbers of retroelements and other repetitive sequences [30], [31]. These two studies suggest that L1 and other retroelements are not causing any new retrotransposition events in iPSCs. However, whole genome sequencing may have limitations in detecting copy number variation of retroelements like L1 and other repetitive sequences due to short sequencing reads, sequencing depth differences between samples and alignment issues of repetitive DNA [31], [32]. Thus, we investigated endogenous L1 retrotransposition in iPSCs through a novel sequencing strategy that we developed. It targets the most active L1 subfamily (L1Hs) and starts from the 3′ end of L1Hs and continues to the genomic sequence, allowing the detection of the polyA tail. The detection of a polyA tail increases the possibility of confirming true retrotransposition events by PCR as it is a key signature of retrotransposition as observed by others [22], [33]. Our results indicate that L1 transcription is activated in iPSCs and that L1Hs retrotransposes in iPSCs at low levels, resulting in a low frequency of somatic insertions.

## Materials and Methods

### Cell culture

The IMR90 cell line (fetal lung fibroblasts; CCL-186) was obtained from the American Type Culture Collection. The NHDF1 cell line (neonatal human dermal fibroblasts) which was originally obtained from Lonza (Allendale, NJ) was a gift of Dr Lowry (University of California at Los Angeles) [34]. The human fetal fibroblasts (HFF) were isolated from fetal foreskin tissues and were previously used to generate iPSCs in the lab [35]. IMR90 and HFF cells were maintained in fibroblast medium: DMEM supplemented with 10% fetal calf serum, glutamine and non-essential amino-acids (Life Technologies, Carlsbad, CA, USA). NHDF1 cells were maintained as described by Lowry et al [34]. The H1 human embryonic stem cell (H1-hESC) line was obtained from WiCell (Madison, Wisconsin, USA) and was maintained in mTeSR1 medium (Stem Cell Technologies, Vancouver, BC, Canada) on matrigel (BD Biosciences, San Jose, CA, USA) coated plates.

### Generation of iPSCs

The cDNA of the reprogramming factors *OCT4, C-MYC, SOX-2*, *LIN28* and *KLF4* were either cloned in the pMX (murine γ-retroviral vector) or FRh11 (a modified FG12 lentiviral vector) vector [35]. Viral stocks were prepared individually by the calcium phosphate precipitation method. Viral stocks were collected 48 and 72 h post-transfection, filtered on a 0.22 µM filter, concentrated and resuspended in Hanks balanced salt solution (Life Technologies, Carlsbad, CA, USA) before being stored at −80°C. Viruses were then normalized for p24gag content by p24 ELISA. IMR90 and HFF derived iPSCs were generated as follows: 5×10^4^ fibroblasts were seeded per well in a gelatin-coated 6 well-plate. The following day, the cells were transduced with the same equivalent of p24 amounts of each viral stock in the presence of 8 µg/mL of polybrene (Sigma-Aldrich, St. Louis, MO, USA) for two hours, after which, the medium was replaced with fresh fibroblast medium and the cells were allowed to expand. Three days post-transduction, 5×10^4^ transduced cells were seeded on an irradiated mouse fibroblasts (iMEFs) feeder layer in a 60 mm dish and cultured in fibroblast medium for a day. The culture medium was then switched to human iPSC medium: Knock-out DMEM (Life Technologies) containing 20% Knockout Serum Replacement (Life Technologies), 2 mM Glutamax (Life Technologies), 0.1 mM non-essential amino acids (Life Technologies), 0.1 mM β-mercaptoethanol (Sigma-Aldrich, St. Louis, MO), and 50 ng/ml of recombinant human basic fibroblast growth factor (Life Technologies). 0.5 mM valproic acid (Sigma-Aldrich) was supplemented for the first 7 days only. Medium was replaced every day for up to three weeks. Around week 3 post-seeding on the feeder layer, iPSC colonies were isolated based on morphological criteria and expanded in mTeSR1 (Stem Cell Technologies) medium on matrigel (BD Biosciences) coated plates. Some clones were then characterized as previously described [35]. The iPSC18 clone derived from human neonatal dermal fibroblasts was generated by Lowry et al [34]. In order to test for L1 expression during the reprogramming process, we initiated reprogramming by transducing HFF with either the FRh11 vector or the pMX γ-retroviral vector encoding the reprogramming factors *OCT4, SOX2, C-MYC* and *KLF4* after which we followed the protocol as described above. At each of the following time point (8, 14, 21 and 28 days post-seeding on the feeder layer), all the cells were trypsinized and collected. Irradiated MEFs were removed from the mixed population by positive selection and only human cells undergoing reprogramming were isolated. RNA was then extracted for each sample by using the RNeasy kit (QIAGEN, Valencia, CA).

### Characterization of iPSCs

Expression of pluripotency gene markers and vector expression silencing assessment have previously been described for the clones used in this study [35]. For teratoma formations, 10^6^ iPSCs (i.e., approximately one 10-cm dish culture) were resuspended in a mixture of DMEM/F12 (Life Technologies) and Matrigel (BD Biosciences) at a ratio of 2∶1. The cell mixtures were then injected intramuscularly into the hind legs of Nod-SCID mice and the animals were monitored once a week. Teratomas were allowed to develop until they would reach approximately 1 cm in size. The animals were then sacrificed and the teratomas were extracted, fixed with 10% formalin, embedded in paraffin, sectioned, and stained with hematoxylin and eosin. The presence of derivative tissues of the mesoderm, ectoderm and endoderm was then confirmed by a pathophysiologist.

### Quantitative real time-RT-PCR

Expression levels of L1 were assessed on total RNA extracts from the different fibroblast cells that were undergoing the reprogramming process or from isolated human iPSC clones and the corresponding parental cells. We used published L1 specific primers and probe previously described [36]. Quantitative real-time RT-PCR was performed by using the iScript one step RT-PCR for probes (Bio-Rad Laboratories, Hercules, CA, USA). In order to normalize for total RNA content, we evaluated the content of GAPDH with the following primers and probe: (S) 5′ GAAGGTGAAGGTCGGAGT 3′, (AS) 5′ GAAGATGGTGATGGGATTTC, (P) 5′ HEX-CAAGCTTCCCGTTCTCAGCC-BHQ-1 3′ (Biosearch Technologies, Novato, CA, USA). Total RNA extracts from iPSC18 clone were a kind gift of Dr Kathrin Plath (University of California at Los Angeles) while the rest of the total RNA samples were isolated using the RNeasy kit (QIAGEN). The Wilcoxon rank sum test was used to assess whether L1 over-expression for each tested sample was significantly higher than that of the parental cells.

### L1Hs library construction and 454 pyrosequencing

Genomic DNA was isolated using the DNeasy Blood & Tissue Kit (QIAGEN). The protocol for L1Hs DNA library preparations was adapted from Ewing et al by using the primers listed below [33]. Briefly, a total of 3.2 µg of genomic DNA from each iPSC clone (hiPSC #7 passage 10, hiPSC #11 passage 12, hiPSC #19 passage 18) and HFF were subjected to the PCR protocol as previously described [33], except that a few modifications were implemented: (1) instead of using the Illumina primers and adapters, 454 primers A and B were used. (2) The library generated from each sample was barcoded differently with a molecular identifier (MID) with the corresponding primers as listed below, to allow the specific marking of the library of each sample before high-throughput sequencing. (3) The 4 libraries were pooled and subjected to 454 high-throughput sequencing using the primer A, which allows reading from the 3′UTR of the L1Hs to the genomic region of insertion, thereby allowing the direct detection of the polyA tail. The same process was used for generating and sequencing L1Hs libraries of the NHDF1 and H1-hESC samples.

### List of primers

L1HsTAILSP1A2 (AC_5931_)
GGGAGATATACCTAATGCTAGATGACAC
A-L16015G MID1 (for HFF)
CGTATCGCCTCCCTCGCGCCATCAGACGAGTGCGTTGCACATGTACCCTAAAACTTAG
A-L16015G MID2 (hiPS #7)
CGTATCGCCTCCCTCGCGCCATCAGACGCTCGACATGCACATGTACCCTAAAACTTAG
A-L16015G MID3 (hiPS #11)
CGTATCGCCTCCCTCGCGCCATCAGAGACGCACTCTGCACATGTACCCTAAAACTTAG
A-L16015G MID4 (hiPS #19)
CGTATCGCCTCCCTCGCGCCATCAGAGCACTGTAGTGCACATGTACCCTAAAACTTAG
A-L16015G MID6 (NHDF1)
CGTATCGCCTCCCTCGCGCCATCAGATATCGCGAGTGCACATGTACCCTAAAACTTAG
A-L16015G MID9 (H1-hESC)
CGTATCGCCTCCCTCGCGCCATCAGTAGTATCAGCTGCACATGTACCCTAAAACTTAG
Primer A
CGTATCGCCTCCCTCGCGCCATCAG
Primer B
CTATGCGCCTTGCCAGCCCGCTCAG
B1-N5TCTGT
CTATGCGCCTTGCCAGCCCGCTCAGNNNNNTCTGT
B2-N5CTTCT
CTATGCGCCTTGCCAGCCCGCTCAGNNNNNCTTCT
B3-N5CTGCA
CTATGCGCCTTGCCAGCCCGCTCAGNNNNNCTGCA
B4-N5TGCCT
CTATGCGCCTTGCCAGCCCGCTCAGNNNNNTGCCT
B5-N5TCTCA
CTATGCGCCTTGCCAGCCCGCTCAGNNNNNTCTCA
B6-N5CAGAG
CTATGCGCCTTGCCAGCCCGCTCAGNNNNNCAGAG
B7-N5TTGAA
CTATGCGCCTTGCCAGCCCGCTCAGNNNNNTTGAA
B8-N5CTTTG
CTATGCGCCTTGCCAGCCCGCTCAGNNNNNCTTTG

### Processing of sequencing data

The resulting 454 DNA sequencing data was first processed as follows before alignment on the human reference genome build 19. Based on the MID sequence barcode, each DNA sequence was assigned its original sample identity i.e. HHF, hiPSC #7, hiPSC #11, hiPSC #19, NHDF1 and H1-hESC. The 454 primers A and B as well as the degenerative sequences were then trimmed for all sequences. By using a specific script for BLAT alignment, the trimmed sequences were then compared to the human reference genome build 19 as described below. The deep sequencing datasets have been deposited in the European Nucleotide Archive (accession number PRJEB6145).

### Identification of reference germline L1Hs insertions

After alignment on the human reference genome, sequences which matched unambiguously annotated L1 insertions from the L1-polyA to the genomic region were identified as reference L1s. These sequences were further categorized as L1Hs or from older families based on the RepeatMasker annotations from the UCSC genome browser, http://genome.ucsc.edu.

### Identification of non-reference L1Hs insertions

Sequences that matched the human reference genome on two unambiguous and distinct locations (i.e. the L1-polyA part of each sequence would align on the L1-polyA of an annotated L1Hs insertion while the rest of the sequence would align on a separate location on the genome) indicated potential non-reference L1 insertions. In order to be considered further, the genomic part of the flanking region of each sequence should display more than 90% identity when compared to the reference genome. (It is to be noted that 80 out of the 100 non-reference germline insertions and all the non-reference somatic insertions displayed more than 95% identity to the reference genome. Out of the 20 remaining germline insertions that displayed an identity percentage between 90% and 94.9%, 14 had already been identified in previous studies and were polymorphic while the remaining six insertions would be specific to that individual). These sequences were used for identification of non-reference germline and somatic insertions.

To identify non-reference germline insertions: Each insertion site (i) either was present in the HFF library (ii) or was previously annotated in any of the five non-reference L1 databases [20], [22], [33], [37]–[39] (iii) or was present in any two iPSCs libraries at these same time (we are assuming that the probability of having the same insertion in two different clones would be very unlikely.) (iv) or was present in a single iPSC library but validated by PCR in HFF (21 out of 22 insertions found in either iPSC clone #7 or #11 only were tested positive in HFF by PCR.) and (v) displayed a polyA tail.

To identify potential somatic insertions in iPSC clones: each non-reference insertion (i) was absent in the HFF library (ii) was present in only one iPSC clone library at a time (iii) was absent from the five published L1 insertion libraries [20], [22], [33], [37]–[39] and the two additional libraries that we generated (NHDF1 and hESC) (iv) displayed negative PCR detection in HFF and (v) displayed a polyA tail.

### Calculation of sequencing depth

The sequencing depth for each sample was calculated based on the average number of sequences detected per reference L1Hs identified.

### PCR validation of non-reference germline L1 insertions

The presence of non-reference germline insertions was verified via site-specific PCR as described by Ewing et al [33]. PCR was performed on 20 ng of HFF DNA template using GoTaq Flexi DNA Polymerase (Promega, Madison, WI, USA) as per manufacturer’s instructions. The 3′ ends and flanking regions of non-reference L1s were amplified with the same AC dinucleotide–specific primer used for the library preparation and a designed reverse primer located near the site of insertion. The specificity of amplification was verified by nested PCR for some insertions as described by Baillie et al [18].

### PCR validation of non-reference somatic L1 insertions

To verify potential somatic insertions in iPSCs, we used the same protocol as described above. PCR yielded negative results in HFF as expected but did not give positive results in the corresponding iPSC clone. We therefore resorted to nested-PCR, a more sensitive method as described by Baillie et al used to identify somatic insertions [18]. We used the same reagents and conditions previously used. For verification of each somatic insertion, we started with 20 ng and then used up to 200 ng of DNA template for each insertion found in each clone. The corresponding amount of HFF DNA was used as a negative control.

### Ethics Statement

This study was approved by the University of California at Los Angeles human Embryonic Stem Cell Research Oversight (ESCRO) committee (ESCRO approval number 2008-008-06) and the University of California at Los Angeles Animal Research Committee (ARC approval number 1993.282.62C).

## Results

### Total L1 expression is upregulated in isolated iPSC clones independent of donors

**Figure 1:**
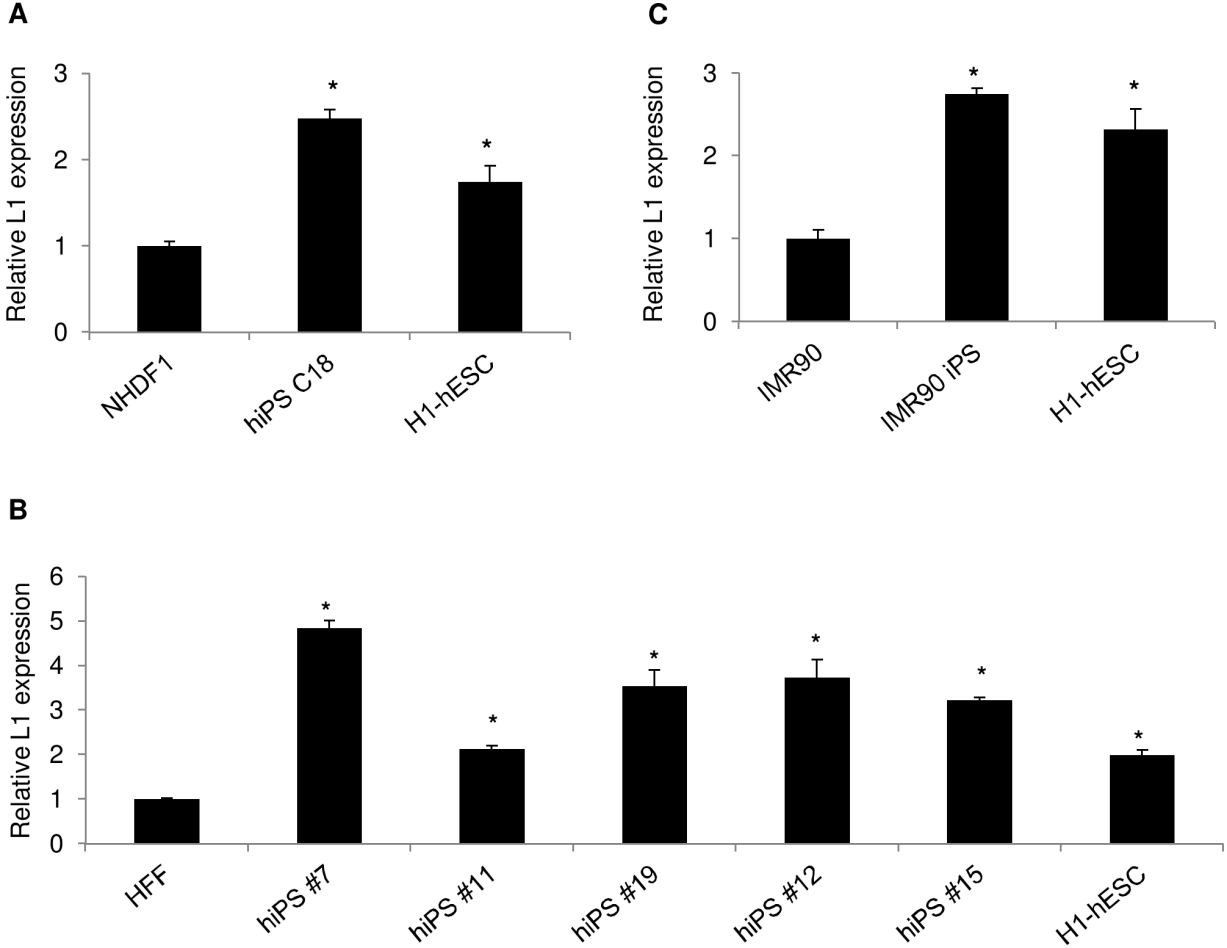
L1 transcriptional up-regulation in human iPSC clones is independent of donors. L1 expression was evaluated by quantitative real-time RT-PCR on total RNA extracted from iPSC clones derived from (A) NHDF1 (B) HFF (C) IMR90 cell line. To evaluate the respective basal level of L1 expression, total RNA extracts from the respective parental cells were subjected to real-time PCR. Real-time RT-PCR results were normalized with respect to GAPDH content. Fold increase of L1 expression was then calculated with respect to the result obtained from the parental cells. Results are shown as average ± standard deviation. RNA extracts from the H1 human embryonic stem cell line was used a positive control. Asterisks denote statistical significant increase in L1 expression when compared to the reference parental cells as assessed by the Wilcoxon rank sum test (p<0.05).

To confirm that L1 is over-expressed as detected by others we assessed total L1 expression in the hiPSC18 clone by using published primers and probe [29], [36]. This clone was derived by Lowry et al from human neonatal dermal fibroblasts 1 (NHDF1) by forced expression of the cDNA of the five reprogramming factors *OCT4, SOX2, C-MYC*, *NANOG*, and *KLF4* from the γ-retroviral vector pMX [34]. RNA extracts were obtained from the hiPSC18 clone as well as from the parental NHDF1 to assess the original L1 basal level of expression. As a positive control, we used RNA extracts from the H1 human embryonic stem cell line (H1-hESC), previously shown to express L1 RNA [40]. As shown in Figure 1A, we observed that the level of L1 expression was around two fold higher in H1-hESC than that of NHDF1, thereby confirming stronger regulation of L1 expression in differentiated cells than in undifferentiated ones. We also detected a 2.5 fold increase in L1 expression in the hiPSC18 clone when compared to that of the parental cells. Interestingly, L1 expression was higher than that found in H1-hESC. To address the possibility of a donor specific-response, we also assessed L1 expression in several iPSC clones derived from fibroblasts from two additional donors. We had previously derived several iPSC clones from human fetal fibroblasts (HFF) by forced expression of the cDNA of the four reprogramming factors *OCT4, SOX2, C-MYC* and *KLF4* encoded by the FRh11 lentiviral vector [35]. These clones express typical hESC markers, have ectopic reprogramming factors expression silenced [35] and are able to form teratomas in mice (Figure S1A, B). As shown in Figure 1B, all iPSC clones showed a 2.1–5 fold increase in L1 transcription level when compared to that of the parental HFF. With the exception of the hiPS #11 clone, these levels were all well above that observed in H1-hESC. We also tested a third iPSC clone that we derived from the IMR90 cell line, a cell line previously shown to support reprogramming [41]. As shown in Figure 1C, we observed that there was a 2.8 fold increase in the IMR90 iPSC clone versus the parental IMR90 cells. The level of L1 expression in this iPSC clone was also higher than that of H1-hESC. Thus, the results obtained in isolated iPSC clones from three independent sources indicated that the observed increase in L1 expression was an intrinsic feature of iPSC clones which did not depend on the donor from whom the cells were obtained. These results confirmed those of a previous study where it was shown that L1 was over-expressed in iPSCs due to L1 promoter derepression as a result of its demethylation [29].

### Total L1 expression is up-regulated during the reprogramming process

**Figure 2:**
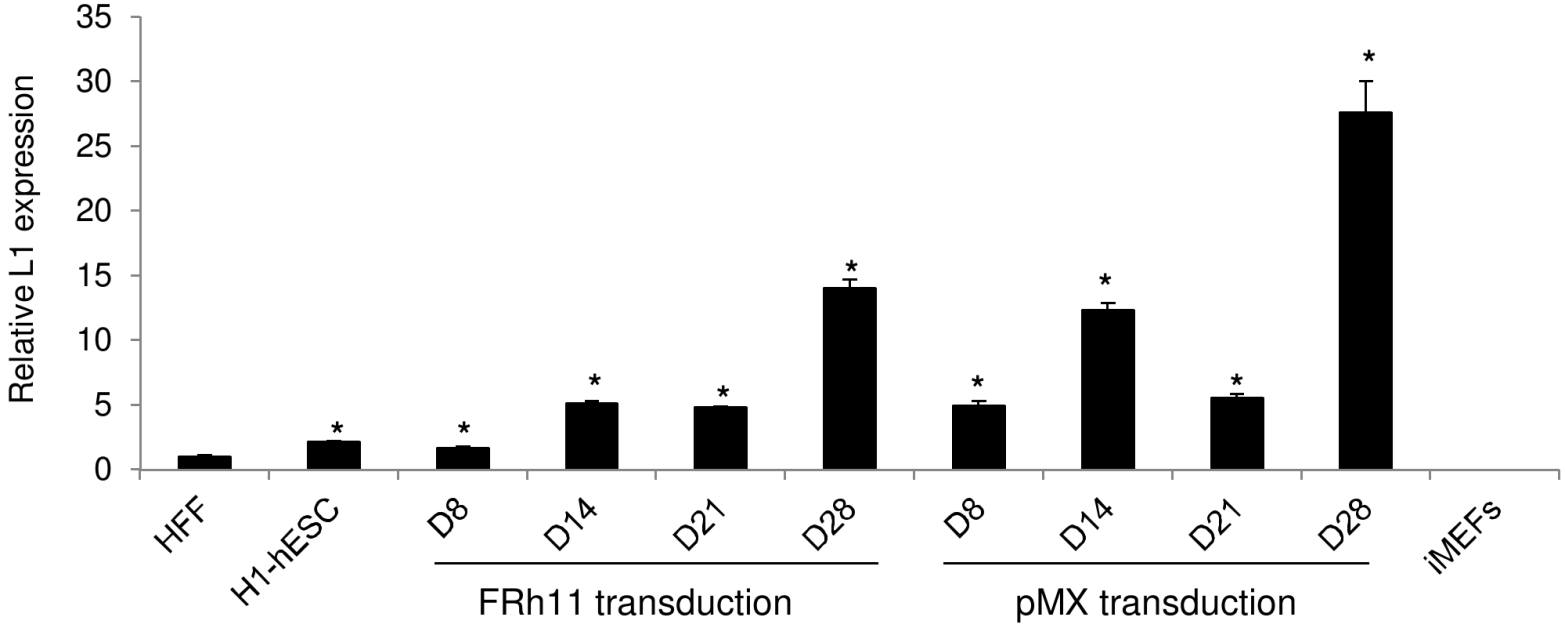
L1 up-regulation is observed during the reprogramming process and is independent of the transducing vector. HFF were transduced with either the FRh11 lentiviral vector or the pMX murine γ-retroviral vector encoding *OCT4, C-MYC, SOX2* and *KLF4*. Three days post-transduction, the cells were then seeded onto a feeder layer of iMEFs and cultured under hESC conditions. Total cells were collected at 8, 14, 21 and 28 days post-seeding and iMEFs were removed by positive selection. Total RNA extracts were obtained from the remaining human cells which were then subjected to quantitative real-time RT-PCR to assess L1 expression. Total RNA extracts obtained from H1-hESC and iMEFs were used as positive and negative controls, respectively. Quantitative real-time RT-PCR results were normalized with respect to GAPDH content. Fold increase of L1 expression was then calculated with respect to the results of HFF. Results are shown as average ± standard deviation. Asterisks denote statistical significant increase in L1 expression when compared to the reference parental cells as assessed by the Wilcoxon rank sum test (p<0.05).

Next, we investigated whether the increase in L1 expression was triggered during the reprogramming process. The cDNA of the four reprogramming factors *OCT4, SOX2, C-MYC* and *KLF4* were expressed from either the FRh11 vector as used in our previous experiments or the pMX vector as used to generate the hiPSC18 clone [34], [35]. We followed a standard protocol for generating human iPSCs [35]: HFF were transduced with either the FRh11 lentiviral vector or the pMX γ-retroviral vector expressing the cDNA of the four reprogramming factors. The transduced HFF were then transferred onto an irradiated mouse embryonic fibroblasts (iMEFs) feeder layer and cultured under hESC culture conditions. By following this protocol, typical human embryonic stem cell-like (hESC-like) colonies which are potential iPSC colonies, are visible around day 21. We thus investigated whether total L1 over-expression was a progressive process during reprogramming or whether it would become apparent only around the time hESC-like colonies would appear (around day 21). Total cells were therefore collected at different time points (8, 14, 21 and 28 days post-seeding on the feeder layer) for RNA extraction. The irradiated mouse embryonic fibroblasts were first removed from the mixed population by positive selection and only human cells undergoing reprogramming were isolated for RNA extraction to assess L1 expression. As shown in Figure 2, on day 8 post-seeding, the L1 expression was about 1.7 fold higher than the basal level when transduction is mediated by the FRh11 vector. The level of L1 expression continued to increase to 4.9–5.1 folds on days 14 and 21 when compared to HFF. The highest level of L1 expression was achieved on day 28 with a 14.1 fold increase over HFF. Interestingly, when the four reprogramming factors were introduced by the pMX γ-retroviral vector, a 5, 12.3, 5.5 and 27.6 fold increase of L1 expression was detected on days 8, 14, 21 and 28 respectively. The reason for the decrease in L1 expression from day 14 to day 21 in that case and a subsequent increase on day 28 is unclear. The increase on day 28 could be due to the fact that at that point, all the cells have become transformed cells but this requires further investigation. During reprogramming, the majority of cells would become transformed cells and not potential iPSCs. However, interestingly, the level of L1 expression on day 21, when we would normally isolate hESC-like colonies for iPSC characterizations and culture, was similar to that found in isolated cultured clones derived from the same parental cells by using four reprogramming factors encoded by the lentiviral vector FRh11, thus supporting our observed results in isolated clones (Figure 1). Our results therefore suggested that L1 over-expression was activated during the reprogramming process by forced expression of four reprogramming factors and was independent of the vector used to introduce these factors. Overall, our data indicated that L1 over-expression is a general phenomenon which is triggered during the reprogramming process and is then maintained in isolated clones.

### A novel high throughput sequencing strategy to detect genome wide L1Hs insertions

**Figure 3:**
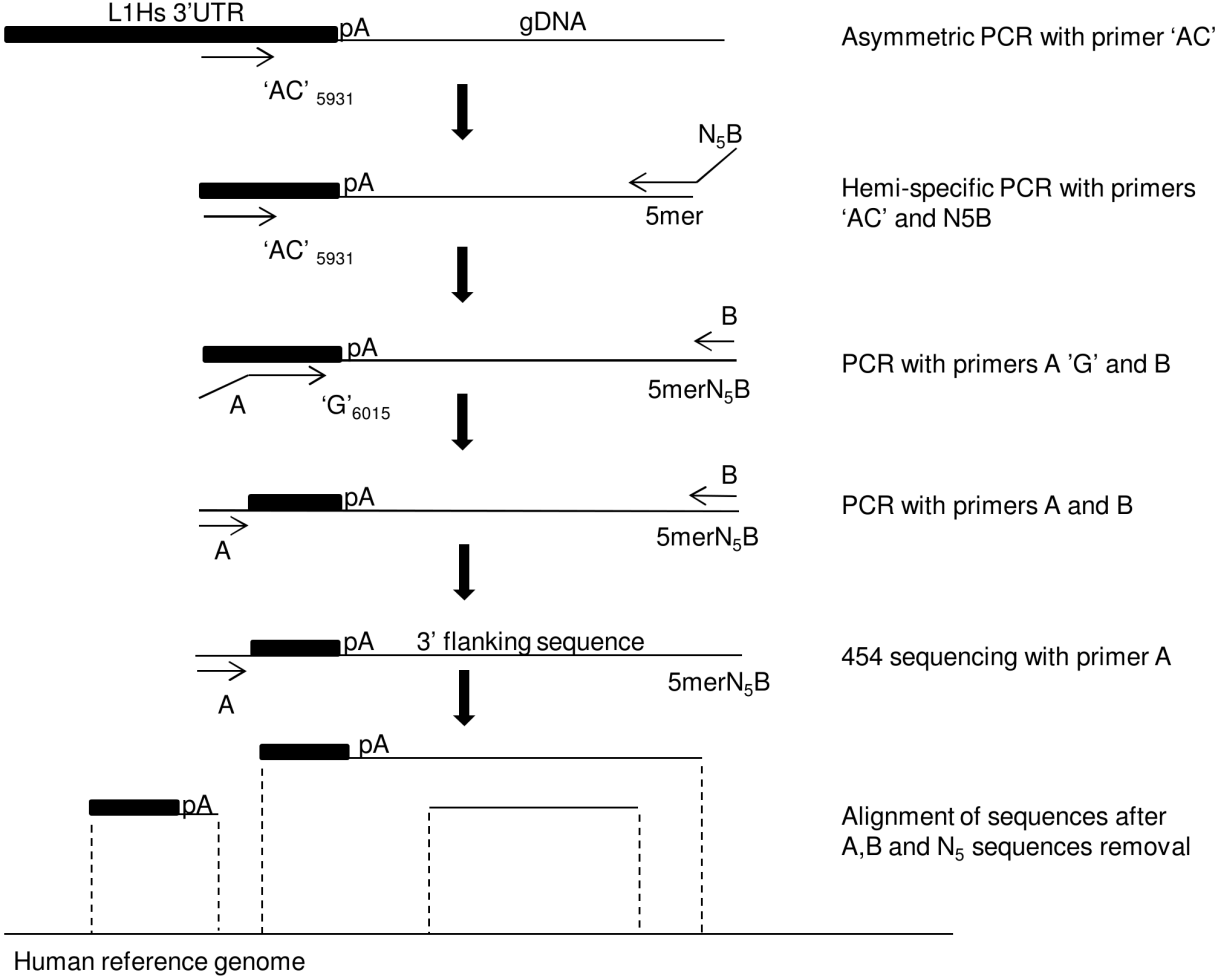
Schematic of PCR strategy for template preparation for 454 sequencing of L1Hs family members (adapted from Ewing et al) [33]. L1Hs libraries were prepared as previously described, except that the 454 primers A and B were used instead of Illumina adapters and that high throughput sequencing was performed by using the primer A instead of the primer B, thus allowing the detection of the polyA (pA) sequence followed by the sequence of the new locus of insertion. The sequences were then processed for mapping on the genome to detect reference as well as non-reference L1Hs insertions. L1Hs reference insertion sequences would match the reference genome from their 3′UTR sequence to the end of their flanking sequence in one location only while non-reference insertion sequences will have their 3′UTR sequence and flanking sequence match the genome on two distinct locations.

Recently, through whole genome sequencing, it was shown that retroelements such as L1 and other repetitive sequences have stable copy numbers in mouse and human iPSCs [30], [31]. However, detecting copy number variation of repetitive sequences is challenging due to sequencing depth differences between samples, short sequencing reads and alignment issues of repetitive sequences [31], [32]. Another group showed that an ectopic engineered reporter L1 which expresses L1 either under an enhancer or a ubiquitous promoter, retrotransposes at higher frequencies in human iPSCs than in the corresponding parental cells, leading the authors to conclude that reprogramming could activate endogenous L1 mobility in iPSCs [29]. However, the engineered L1 differs from endogenous L1 in the use of enhancers and ubiquitous promoters to overexpress L1 reporter RNA. In addition, it may not recapitulate local regulation of L1 retrotransposition which would affect genomic location of insertion. Furthermore, the number of engineered L1 introduced per cell by nucleofection is unknown and may affect the number of retrotransposition events per cell. We thus investigated endogenous L1 retrotransposition activity in human iPSCs by using a sensitive targeted high-throughput DNA sequencing method. We reasoned that new L1 insertions would be the result of retrotransposition events from the L1Hs subfamily, the youngest and most active L1 subfamily in humans [13], [15], [19]. We therefore adapted the L1Hs library constructions previously developed for high throughput sequencing through the Illumina platform to detect germline as well as new somatic L1Hs insertions [21], [33]. The L1Hs subfamily contains the ‘AC’ and ‘G’ nucleotides characteristics in their 3′ end that could be used to distinguish them from other subfamilies [13], [33]. By using the ‘AC’ and ‘G’ primers, we therefore generated a library of L1Hs sequences for each sample tested as previously described [33]. However, we brought two major changes to the previous approach (Figure 3). Firstly, we adapted our libraries for 454 sequencing by replacing the Illumina adapter sequences by the 454 sequencing primers A and B. We reasoned that the longer reads from 454 sequencing platform would enable more accurate mapping and reduce false positives. Secondly, we used a novel sequencing strategy. Instead of conventional sequencing with the primer B, which allows reading from the genomic sequence of the new locus of insertion to the 3′ untranslated region (UTR) of L1Hs as done by others, we sequenced in the opposite direction by using the primer A (Figure 3) [22], [33]. We reasoned that direct sequencing of the junction between the inserted L1Hs and the new locus of insertion would significantly reduce the possibility of false positives as this would allow the direct detection of the polyA sequence at the end of the 3′UTR, one of the known hallmarks of retrotransposition (Figure 3). Previous studies have shown that the level of PCR confirmation of new insertions is significantly higher whenever the polyA sequence can be detected [22], [33]. To validate our sequencing strategy, we first focused on identifying non-reference germline insertions.

### Detection of germline L1Hs insertions

**Figure 4:**
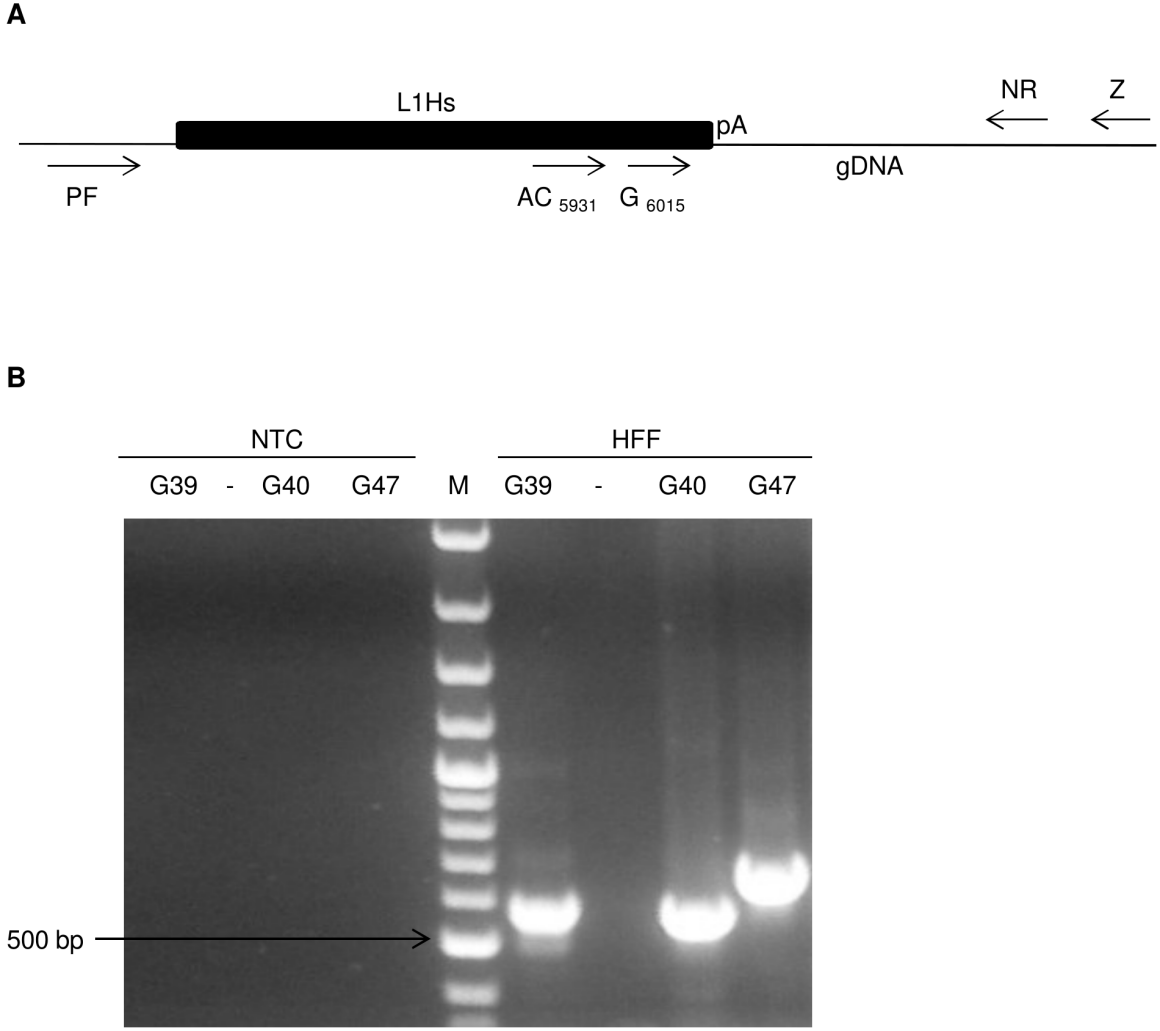
PCR validation of non-reference L1Hs PCR validation. General PCR strategy to verify non-reference germline and somatic L1Hs insertions is shown. (a) DNA fragment are amplified with the primer AC_5931_ located in L1Hs and the reverse primer Z located near the new locus of insertion. To confirm our results, some of these fragments were subjected to nested-PCR by using the internal primers G6015 and NR. Primers PF were used to verify amplification of empty sites. (b) Typical results of L1Hs confirmed in HFF by the AC_5931_ and Z primers are shown. The arrow shows the 500 bp band of the 100 bp ladder (M). Unnecessary lanes were removed.

It has previously been shown that each individual possesses approximately 800 germline L1Hs insertions [21], [33]. In order to validate our new sequencing strategy, we verified our ability to recover these germline insertions. L1Hs libraries from hiPSC #7, hiPSC #11, hiPSC #19 clones as well as the parental cells HFF, were thus subjected to 454 deep sequencing. Through deep coverage sequencing, we identified a total of 737 germline L1Hs out of approximately 800 possible insertions, thus showing that our method is efficient in capturing L1Hs (Table 1, Table S1). Of these 737 germline L1Hs, we detected a total of 637 reference L1Hs already annotated in the human reference genome build 19 while the remaining 100 germline insertions detected were not previously annotated (Table 1, Table S2). These non-reference germline insertions all had a poly A tail located after the L1 3′UTR. Of these insertions, 77 were found in intergenic regions while the remaining 23 insertions were found in genes, exclusively in introns (Table S2). We also verified whether some of our insertions could be found in other published L1 databases, which would be an additional indication of successful capture of non-reference insertions. We observed that 24 of the non-reference germline insertions were unique to the individual from whom HFF were isolated while the remaining 76 insertions were found in at least one of the five published non-reference L1 insertion databases [20], [22], [37]–[39], indicating their polymorphic nature (Table S2). Furthermore, PCR had ∼94% (58/62) success rate for confirming the presence of these germline insertions (Figure 4A, 4B, Table S3), a success rate similar to that found in other studies [22], [33]. Specificity of DNA amplification was confirmed by nested PCR for some insertions (Table S3). Taken together, these results show that the method of Ewing et al can be easily adapted for 454 sequencing and that our novel sequencing strategy reliably detected germline insertions [21], [33].

**Table 1:** Summary of the germline insertion results.

Samples	Total numberof reads	Total numberof L1Hsdetected	Total number ofreference L1Hs	Total numberof non-referenceL1Hs	Averagesequencingdepth	PCRvalidation(58/62)
HFF, hiPSC #7,#11 & #19	222 350	737	637	100	38.7x	94%

### Potential somatic insertions detected in human iPSCs

Having validated the detection of non-reference germline insertions in HFF, we then addressed the issue of whether the two iPSC clones #7 and #11 derived from HFF harbored somatic L1 insertions as a result of L1 over-expression. We did not take into account any potential somatic insertions from a third iPSC clone derived from HFF (hiPSC #19 clone) since it gave rise to only ectodermic tissues during in vivo iPSCs differentiation into teratomas and therefore may not be a fully reprogrammed clone (Figure S1c). We identified a total of seven unique, potential somatic insertions: four in the hiPSC #7 clone and three in the hiPSC #11 clone (Table 2, Table S4). Four of the seven potential insertions were in genes, exclusively in introns, while the remaining three were in intergenic regions. A few observations highly suggested that these insertions were somatic. Each of these different insertions had the expected polyA tail adjacent to the polyA signal sequence (Table S4). In addition, all of these insertions were absent from the dbRIP database as well as from four other non-reference L1 databases [20], [22], [37]–[39]. They therefore do not represent any known L1Hs germline polymorphisms. Furthermore, as expected, when we tested the presence of each of these insertions in HFF by PCR, they were all absent (data not shown). Taken together, these observations indicated that the insertions were somatic. We then verified evidence of amplification of these insertions in iPSCs. However, we could not detect them in the corresponding hiPSC #7 and hiPSC #11 clones by regular PCR. The amplifications were still negative when PCR reagents and primers were changed or when we resorted to nested PCR with different primers and using ten times more iPSC DNA multiple times. As expected, these insertions could not be amplified in HFF (data not shown). We successfully amplified the empty sites, indicating that the primers were capable of amplification (data not shown). Therefore, it is likely that these insertions are in low abundance in iPSCs without amplification and remain at the same low abundance in the cell population such that they could not be detected by PCR. In support of this, we observed that there was a difference between the average sequencing read counts for germline insertions and that of the potential somatic insertions. The somatic insertions had an average of read count of one per sample (Table S4) while germline insertions (Table S2) had average read count of 12.4 per sample, potentially suggesting a low abundance of somatic insertions. Being of low read count, we addressed the possibility of these insertions being sequencing artifacts. We reasoned that artifacts should be present in both the germline and somatic insertion datasets and that they would have a read count of one. We checked 26 germline insertions with a read count of one by PCR. These 26 insertions were all germline insertions since 24 of them could be detected positively by PCR in HFF while the remaining two that could not be detected in HFF were both found in published germline L1Hs non-reference databases. As such, none of the 26 sequences of read count one was due to sequencing artifacts.

**Table 2:** Summary of the somatic insertion results.

Insertion	Present in iPSC#	Chromosome	Strand	Start	End	Gene	Intron/Exon
S11-1	11	chr5	−	41234256	41234440	C6	Intron
S11-2	11	chr8	+	114687635	114687886	intergenic	
S11-3	11	chr9	+	14256638	14256747	NFIB	Intron
S7-1	7	chr1	+	223488996	223489401	SUSD4	Intron
S7-2	7	chr7	+	99584283	99584487	intergenic	
S7-3	7	chr12	+	40281021	40281040	SLC2A13	Intron
S7-4	7	chr13	+	40558942	40559150	Intergenic	

The high rate of positive detection for read count of one for germline insertions (24/26 i.e. more than 92%) was similar to those previously reported for germline insertions [22], [33]. This allowed us therefore to estimate that less than 8% of insertions with read count of one could not be detected either due to the presence of contaminants or PCR failure. The same low percentage of PCR failure and/or contaminants should then also be present in the somatic insertions as well. This in turn supports the fact that the somatic insertions detected are unlikely artifacts.

Next, we further addressed the possibility of these insertions being germline insertions originating from contaminating DNA. We had already verified that the potential somatic insertions were absent from five different databases of non-reference germline insertions derived from at least 80 individuals (the dbRIP and the four other published databases) [20], [22], [33], [37]–[39]. We further verified the absence of these insertions in two additional datasets of germline L1Hs sequences derived from NHDF1 and H1-hESC (Table S5). In summary, the somatic insertions were absent from seven germline databases. They are therefore unlikely due to the presence of germline L1Hs derived from contaminants. Thus, we conclude that L1Hs retrotransposes in iPSCs at low levels and iPSCs contain L1Hs somatic insertions in low abundance.

## Discussion

Assessing genomic stability of iPSCs is of utmost importance before their use in regenerative medicine. Here, we showed that overexpression of the endogenous mutagen L1 was triggered during reprogramming and that overexpression was sustained in isolated iPSC clones later as previously shown [29]. Through a novel sequencing strategy, we also identified seven potential somatic L1Hs insertions in two iPSC clones with a low read count. Our study therefore indicates that L1Hs does retrotranspose at low levels in human iPSCs as previously reported with an exogenous engineered reporter L1 [29].

High throughput sequencing of the L1Hs subfamily resulted in the identification of seven potential L1Hs somatic insertions in two iPSC clones. There are several indications that our insertions were new somatic retrotranposition events: (1) they all had a polyA tail sequence, a key signature of retrotransposition as observed by others [22], [33], (2) they were all absent in the HFF when tested by PCR, (3) each insertion was different and unique to each iPSC clone, and (4) they were all absent from seven L1 insertion databases and are therefore unlikely to be polymorphic insertions resulting from contaminating DNA. (5) The high rate of positive detection of germline insertions (>92%) with read counts of one is a strong indication that the detection process is successful. Our somatic insertions are thus not false positives acquired during the deep sequencing process but are likely to be true somatic events in iPSCs. These insertions could be unambiguously located on the human reference genome unlike insertions into repetitive regions where their exact location insertion is impossible to assess [20]. Therefore the true number of somatic insertions due to L1 retrotransposition could be higher.

Why could we not positively detect these seven somatic insertions in the corresponding iPSC clone? Our high rate of PCR validation in confirming the total germline insertions indicates that our validation approach is successful in confirming new insertions (∼94% success), which would predict PCR confirmation for about 6 of our 7 somatic insertions. One explanation for not detecting these insertions is low abundance in the culture at the time of DNA extraction. Germline retrotransposition insertions are present in all cells whereas somatic retrotransposition insertions occur spontaneously at any time resulting in mosaicism [42]–[46]. When taken as a bulk population, different cell/tissue samples having undergone somatic retrotransposition would have variable and low numbers of cells harboring somatic insertions. Assuming that iPSCs with these somatic insertions grow at the same rate as other iPSCs, this low frequency would be maintained in the population. While being detected once by sensitive high throughput sequencing analyses, these rare somatic insertions would be unlikely to be detected again in each of the iPSC DNA sample and hence, their low read count and the inability to detect them by PCR.

Our results are consistent with those of a previous study where L1 mobility was detected in iPSCs using an exogenous L1 reporter but are in contrast to those of two other studies which showed stable number of repetitive sequences such as L1 in human or mouse iPSCs by whole genome sequencing [29]–[31]. However, detection of possible copy number variation of repetitive sequences by whole genome sequencing has limitations [32]. The difference in results between our study and others may be explained by the fact that our method which targets L1Hs could be more sensitive than whole genome sequencing in detecting low abundance L1Hs. Our results therefore underscore the use of sensitive methods to detect genomic variants in iPSCs which may be found at low levels.

## Conclusion

Our findings suggest that endogenous L1Hs are capable of retrotransposition in human iPSCs, albeit in low numbers and that these cells harbor somatic insertions at low levels. Our work highlights the importance of careful examination of human iPSCs to detect any possible L1 insertion that may lead to adverse effects.

## Supporting Information

Figure S1 **iPSC clones can form teratomas with the 3 distinctive germ layers.** Approximately 10^6^ iPSC cells were resuspended in a mixture of DMEM/F12 and matrigel. The cell mixtures were then injected intramuscularly into the hind legs of Nod-SCID mice and teratomas allowed to develop until they reach approximately 1 cm in size. The teratomas were then extracted and fixed with 10% formalin. Then they were embedded in paraffin, sectioned, and stained with hematoxylin and eosin. Tissues derived from the mesoderm, ectoderm and endoderm were confirmed by a pathophysiologist. Results are shown for (A) hiPSC #7 (B) hiPSC #11 and (C) hiPSC #19.
(TIF)

Table S1 **Summary of total sequences obtained after 454 sequencing and depth coverage.**
(XLSX)

Table S2 **Non-reference germline L1Hs detected in HFF.**
(XLSX)

Table S3 **PCR validation results and primers for non-reference germline L1Hs in HFF.**
(XLSX)

Table S4 **Non-reference potential somatic L1Hs detected in iPSCs.**
(XLSX)

Table S5 **Non-reference germline L1Hs detected in NHDF1 and hESC.**
(XLSX)
