# Supplementary figures and images for: Deep Sequencing Reveals Low Incidence of Endogenous LINE-1 Retrotransposition in Human Induced Pluripotent Stem Cells
Source: PLoS One. 2014 Oct 7;9(10):e108682. doi: 10.1371/journal.pone.0108682 (PMC4188539; doi:10.1371/journal.pone.0108682)

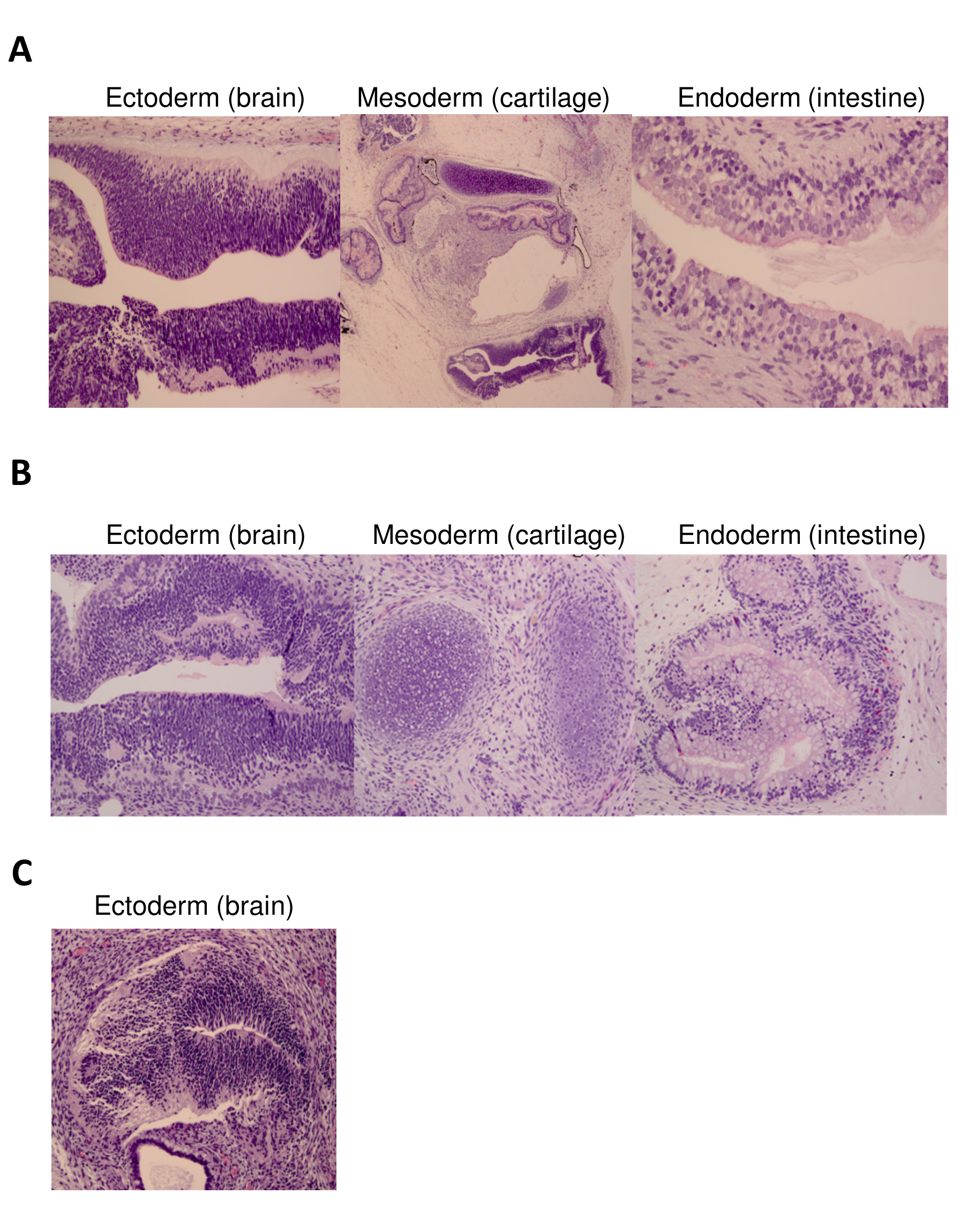

Supplement: Figure S1 — iPSC clones can form teratomas with the 3 distinctive germ layers. Approximately 106 iPSC cells were resuspended in a mixture of DMEM/F12 and matrigel. The cell mixtures were then injected intramuscularly into the hind legs of Nod-SCID mice and teratomas allowed to develop until they reach approximately 1 cm in size. The teratomas were then extracted and fixed with 10% formalin. Then they were embedded in paraffin, sectioned, and stained with hematoxylin and eosin. Tissues derived from the mesoderm, ectoderm and endoderm were confirmed by a pathophysiologist. Results are shown for (A) hiPSC #7 (B) hiPSC #11 and (C) hiPSC #19. (TIF) [file pone.0108682.s001.tif]
